# A walk through tau therapeutic strategies

**DOI:** 10.1186/s40478-019-0664-z

**Published:** 2019-02-15

**Authors:** Santosh Jadhav, Jesus Avila, Michael Schöll, Gabor G. Kovacs, Enikö Kövari, Rostislav Skrabana, Lewis D Evans, Eva Kontsekova, Barbara Malawska, Rohan de Silva, Luc Buee, Norbert Zilka

**Affiliations:** 1 Institute of Neuroimmunology, Slovak Academy of Sciences, Dubravska 9, 845 10 Bratislava, Slovakia; 2 Centro de Biologia Molecular “Severo Ochoa”, Consejo Superior de Investigaciones, Cientificas, Universidad Autonoma de Madrid, C/ Nicolas Cabrera, 1. Campus de Cantoblanco, 28049 Madrid, Spain; 3 Networking Research Center on Neurodegenerative, Diseases (CIBERNED), Instituto de Salud Carlos III, Madrid, Spain; 4 Wallenberg Centre for Molecular and Translational Medicine, University of Gothenburg, Gothenburg, Sweden; 5 Department of, Psychiatry and Neurochemistry, University of Gothenburg, Gothenburg, Sweden; 6 Clinical Memory Research Unit, Department of Clinical Sciences, Lund University, Malmö, Sweden; 7 Dementia Research Centre, University College London, London, UK; 8 Institute of Neurology, Medical University of Vienna, AKH 4J, Währinger Gürtel 18-20, 1097 Vienna, Austria; 9 Department of Mental Health and Psychiatry, University Hospitals of Geneva, Geneva, Switzerland; 10 AXON Neuroscience R&D Services SE, Dvorakovo nabrezie 10, 811 02 Bratislava, Slovakia; 11 Gurdon Institute and Department of Biochemistry, University of Cambridge, Cambridge, CB2 1QN UK; 12 Department of Physicochemical Drug Analysis, Faculty of Pharmacy, Jagiellonian University Medical College, Medyczna 9, 30-688 Cracow, Poland; 13 Reta Lila Weston Institute and Department of Clinical and Movement Neurosciences, UCL Queen Square Institute of Neurology, 1 Wakefield Street, London, WC1N 1PJ UK; 14 Universite of Lille, Inserm, CHU-Lille, UMRS1172, Alzheimer & Tauopathies, Place de Verdun, 59045 Lille cedex, France

**Keywords:** Alzheimer’s disease, Tau vaccines, Therapeutic interventions, Immunotherapy, Tauopathies, PET imaging, Aggregation

## Abstract

Tau neuronal and glial pathologies drive the clinical presentation of Alzheimer’s disease and related human tauopathies. There is a growing body of evidence indicating that pathological tau species can travel from cell to cell and spread the pathology through the brain. Throughout the last decade, physiological and pathological tau have become attractive targets for AD therapies. Several therapeutic approaches have been proposed, including the inhibition of protein kinases or protein-3-O-(N-acetyl-beta-D-glucosaminyl)-L-serine/threonine Nacetylglucosaminyl hydrolase, the inhibition of tau aggregation, active and passive immunotherapies, and tau silencing by antisense oligonucleotides. New tau therapeutics, across the board, have demonstrated the ability to prevent or reduce tau lesions and improve either cognitive or motor impairment in a variety of animal models developing neurofibrillary pathology. The most advanced strategy for the treatment of human tauopathies remains immunotherapy, which has already reached the clinical stage of drug development. Tau vaccines or humanised antibodies target a variety of tau species either in the intracellular or extracellular spaces. Some of them recognise the amino-terminus or carboxy-terminus, while others display binding abilities to the proline-rich area or microtubule binding domains. The main therapeutic foci in existing clinical trials are on Alzheimer’s disease, progressive supranuclear palsy and non-fluent primary progressive aphasia. Tau therapy offers a new hope for the treatment of many fatal brain disorders. First efficacy data from clinical trials will be available by the end of this decade.

## Introduction

Tau protein is considered to be one of the most peculiar proteins in the central nervous system. It is located in several cell compartments, including the axon, dendrites, nucleus, nucleolus, cell membrane and synapses [310]. However, tau is also present in the interstitial fluid [284, 370], and can pass into cerebrospinal fluid (CSF), where it is found at concentrations of 10-25 pg/ml (pT181-tau) or 300-400 pg/ml (tau) [28, 29, 248]. In physiological conditions, extracellular tau may enter neurons either via a dynamin-mediated endocytic mechanism or by classical endocytosis [95]. In neurodegenerative tauopathy, diseased modified tau can propagate along neuroanatomically connected brain areas via multiple mechanisms and spread tau pathology throughout the brain [231].

Tau belongs to the group of natively disordered proteins, which exist in a highly flexible, unfolded structural state, largely devoid of well-defined secondary and tertiary structure, although they are able to fold after binding to targets [329]. The highly flexible structure of tau protein allows interaction with multiple partners, suggesting its involvement in numerous signalling pathways [308]. The dark side of its structural repertoire is its ability to interact with other tau molecules to form oligomers and filaments [298, 338, 339]. These complexes cause degeneration of neurons and glial cells [97], manifesting as a group of neurodegenerative disorders termed ‘tauopathies’ [312].

The most prominent tauopathy is Alzheimer’s disease (AD), the common cause of dementia in older adults. AD is an incurable, progressive degenerative disease of the brain, characterized by the presence of tau and ß- amyloid (Aß) pathology [286]. There are no disease-modifying drugs available for AD; only symptomatic treatments trying to counterbalance the neurotransmitter disturbance exist. No significant new drug for AD has been approved in the last 14 years, despite extensive clinical trials. The pipeline has been plagued with significant failures, with more than 400 failed clinical trials since the last symptomatic Alzheimer’s drug was approved [71].

Despite the field being aware that tau pathology correlates well with the onset and progression of AD for almost 40 years [39], it is only now that tau targetted therapy has become attractive for clinical trials. A multitude of tau antibodies and vaccines have been tested in preclinical studies in the last two decades. Currently, eight humanised tau antibodies and two tau vaccines have entered clinical trials either for AD or frontotemporal dementia (FTD) [65, 71](www.alzforum.org). In light of the failure of the clinical trials with amyloid targeting drugs, tau therapy is manifesting as the frontrunner in the search for an effective treatment for AD.

## Tour de tau - tau as a protein with multiple faces

In contrast to amyloid precursor protein (APP), the function of tau protein was already known at the time of the discovery of it as a constituent of neurofibrillary degeneration. Tau is a microtubule-associated protein (MAP), promoting the polymerization and assembly of microtubules [351]. In the adult human brain, there are six isoforms of tau protein generated by alternative splicing from a single gene located on chromosome 17 [120, 238]. At the N-terminal end, they differ by the addition of a 29 amino-acid sequence (1 N) or as replicates (2 N - total of 58 amino acids) coded by exons 2 and 3. The sequence coded by exon 3 is only present if the sequence encoded by exon 2 is inserted. Interestingly, the 2 N tau isoforms are weakly expressed in the human brain [119, 214, 295]. The microtubule binding region (MTBR), has three (3R: R1, R3, R4) or four repeat domains (4R: R1-R4). The sequence encoded by exon 10 allows the insertion of a 31 amino acid microtubule binding domain (R2) which is inserted after the first repeat R1. Tau isoforms with 3R and 4R are equally expressed, since their ratio is about 1:1 in the human brain [295]. However, some neurons do not express 4R tau isoforms. For instance, granular cells of the dentate gyrus only express mRNAs of 3R-tau isoforms [119]. Thus, tau isoforms have different cellular and laminar distribution in the human brain [46].

The strict classification of tau protein as a MAP may have delayed research on its other biological functions. If sequence homology (70-90%) with other MAPs is evident in the microtubule binding domains, the N-terminal portion of tau is unique. It must therefore have other unique functions [194]. Logically, as a MAP, tau has functions in cell trafficking, but it also interacts with dynactin and synaptogyrin-3, suggesting specific related-functions, such as synaptic vesicle control [213, 224].

**Fig. 1:**
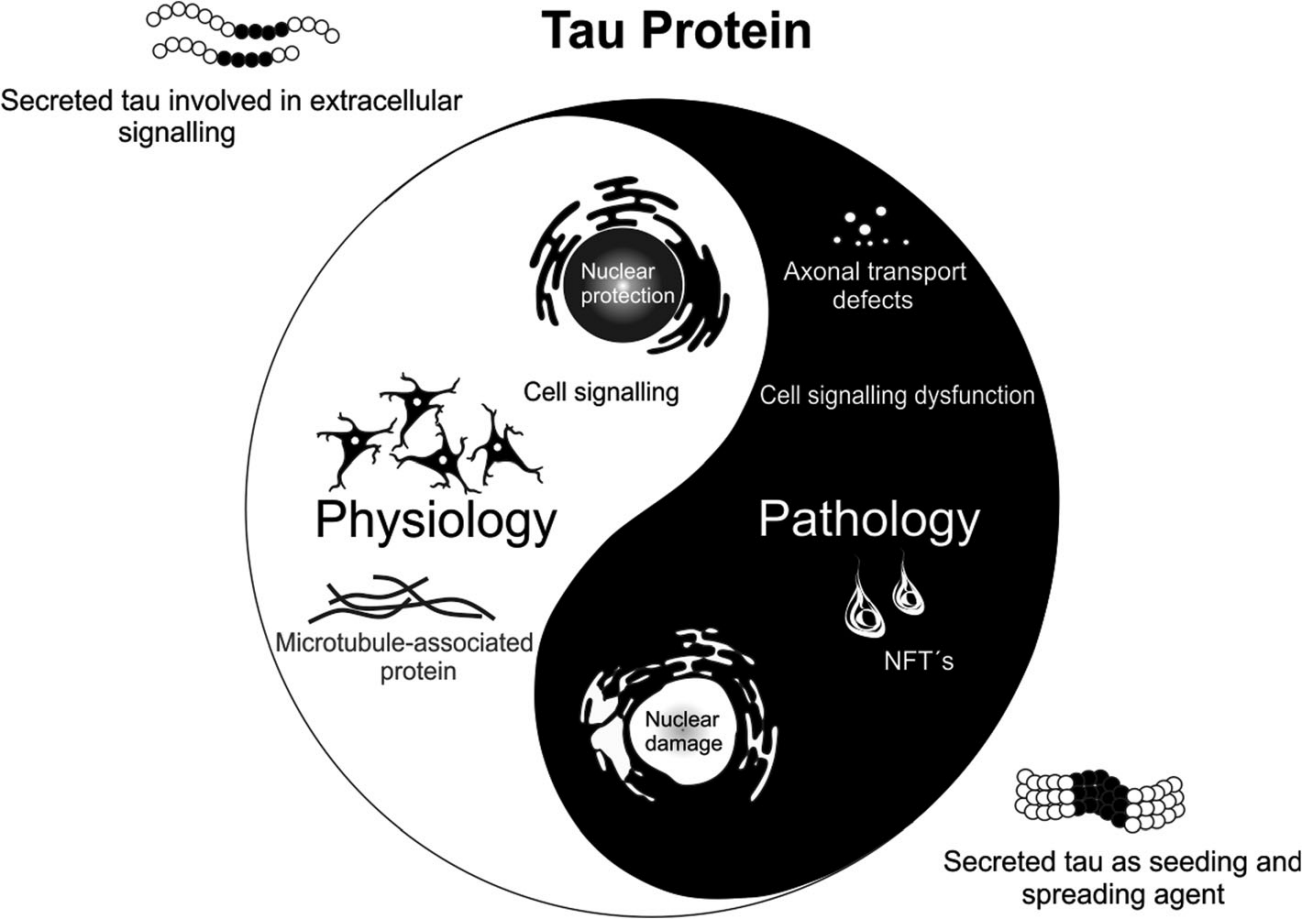
Yin and Yang of Tau protein

The first unexpected functions of tau may be related to its nuclear localization [201]. These initial findings were widely discussed, but nowadays, it is clearly established that tau binds to nucleic acids, and may be involved in chromatin remodelling [53, 104, 146, 252, 266, 267]. The binding of tau to DNA may allow protection against reactive oxygen species [316, 349], and binding to RNA may contribute to ribosome stability and miRNA activity [35]. Altogether, these data strongly suggest that tau may modulate gene expression and RNA stability. Such observations are also supported by tau loss-of-function in pathological conditions. For instance, formation of tau oligomers leads to DNA/RNA damage [337], RNA and ribosome instability [225] and changes in nuclear organization and protein expression [103]. Binding of tau to tRNAs may also initiate tau aggregation by forming droplets through complex coacervation [378]. Moreover, pathological tau can interact with nucleoporins of the nuclear pore complex (NPC) and affect their structural and functional integrity [93] (Fig. 1).

Secondly, tau may also play a role in cell signalling. The longest brain tau isoform with 441 amino acids (aa) has 85 putative sites of phosphorylation. Thus, tau may act as a buffer for cell signalling. For instance, tau may serve as a ‘phosphorylation sink’ for the p25-Cdk5 complex, hence sequestering it away from other death-inducing substrates [130]. Tau may also interfere with tyrosine kinase family Src/Fyn signalling at dendrites [49, 152]. Tau also interacts with phosphatase and tensin homolog (PTEN) and modulates insulin signalling. Recent data suggest that loss of tau function leads to an impaired hippocampal response to insulin, caused by altered insulin receptor substrate 1 (IRS-1) and PTEN activities [218].

Finally, the cytosolic tau protein may also be secreted. This secretion is stimulated by neuronal activity [263]. Such secretion is likely to occur through non-conventional secretory pathways [44]. Recent data suggest that such secretion may be similar to that of fibroblast growth factor 2 (FGF-2), including oligomerization, binding to phospho-inositol, and extracellular capture by heparan sulphate proteoglycans [164]. An alternative pathway is the secretion of pro-interleukin 1, which requires proteolysis. Interestingly, C-terminal-tau fragment Δ422–441 was significantly more secreted than full length tau [261]. Tau is also secreted within extracellular vesicles such as exosomes [346] and ectosomes [89]. In pathological conditions, secreted tau may participate to tau seeding and spread (discussed later).

To sum up, tau has multiple functions in addition to axonal microtubule assembly. All of these recently discovered tau functions may contribute to the development of tau pathology and related events (Fig. 1). These discoveries further strengthen the case for tau as the therapeutic target for AD and tauopathies.

## Tau as a driver of neurodegeneration

AD is a double proteinopathy, characterized by the presence of both tau-reactive neurofibrillary lesions and β-amyloid (Aβ) depositions (senile plaques; SPs). The importance of both proteins, which are present also under physiological circumstances, in the development of AD is extensively debated. Numerous clinicopathological studies were published, favouring both histological lesions, i.e. NFTs and SPs. However, since the early nineties, most studies found a strong correlation between neocortical NFT load and cognitive impairment [94].

The progression of neurofibrillary pathology begins in the entorhinal cortex, in contrast to the spreading of Aβ, where the presence of neocortical SPs precedes the appearance of hippocampal SPs [39, 91, 320, 327]. Aβ pathology is present even in cognitively intact persons, so amyloid deposition is not sufficient to explain the clinical phenotype of AD [77]. In contrast, NFT burden in associative neocortical areas is strongly related with clinically overt dementia. The Braak staging [39] for NFTs, used to define the neuropathological severity of AD in the general neuropathological practice, reveals a strong correlation with cognitive decline [92, 121]. In a study of an oldest-old population, Gold and colleagues [121] found that unlike younger cohorts, Braak stages did not precisely reflect the severity of dementia. Braak stage III correlates poorly with cognitive decline, while Braak stages IV or greater are consistently associated with at least mild dementia. This discrepancy is most likely due to the increasing prevalence of mixed neuropathologies in the oldest-old, such as a combination of vascular lesions and AD pathology [156].

As in all neurodegenerative diseases, AD is characterised by selective vulnerability of specific brain regions, cortical layers, and neuronal populations. The anatomical distribution of tau and neuronal loss reflects the different clinical signs of AD well. Anterograde memory problems at the beginning of the symptomatology are related to tau-burden in the medial temporal lobe [94]. During the progression of the clinical presentation, other signs, such as agnosia, apraxia or speech and behavioural problems will add to the memory problems, corresponding to the involvement of different associative or limbic regions. The neuropathological background for acalculia and visuospatial dysfunction is related to the involvement of tau pathology in the parietal lobe [94]. Ideomotor and dressing apraxia is linked to NFT densities in the anterior cingulate cortex, while constructional apraxia relate to NFT densities in the superior parietal, posterior cingulate and occipital cortex [113]. A significant relationship exists between associative visual agnosia and tau burden in the secondary visual cortex (Brodmann area 18) and the occipitotemporal visual association cortex (Brodmann area 37 and ventral 19) [114]. The high NFT density in the superior parietal cortex (Brodmann area 7), posterior cingulate cortex (Brodmann area 23), and CA1 subfield of the hippocampus plays a role in developing temporo-spatial disorientation [115]. Cases with atypical AD, such as posterior cortical atrophy, also underline the importance of tau pathology in developing clinical signs. Patients presenting mainly with visual symptomatology have a high NFT burden in the occipito-parieto-temporal junction and posterior cingulate cortex [138]. The anterior brain regions are less involved as compared to the “classic” form of AD.

Behavioural problems or speech disorders, more suggestive of other neurodegenerative diseases such as frontotemporal dementia, could also be present in neuropathologically confirmed AD. In contrast, prefrontal syndromes are correlated with atypical distribution of NFTs in the dorsolateral, median and orbitofrontal areas [340]. These clinicopathological observations underline the importance of the tau protein in the pathogenesis of AD and its subtypes (amnestic, dysexecutive/behavioural, visuo-spatial, and language presentation).

Tauopathies are clinically, biochemically and morphologically heterogeneous neurodegenerative diseases characterized by the deposition of abnormal tau (microtubule associated protein tau; MAPT) in the brain. Neuropathological phenotypes are distinguished based on the distinct involvement of anatomical areas, cell type, and presence of distinct isoforms of tau in the pathological deposits [172]. If tau protein deposition is the predominant feature, the term primary tauopathy is used. The nomenclature overlaps with the classification of frontotemporal lobar degeneration (FTLD). Disorders characterized by tau pathologies considered having other (possibly diverse) driving forces (e.g. Creutzfeldt–Jakob disease, Down’s syndrome) are called secondary tauopathies [108].

Tauopathies are distinguished based on the ratio of 3 repeat (3R)- and 4R-tau and two or three major bands (60, 64, and 68 kDa) in Western blot of sarkosyl-insoluble fractions [184, 296, 312]. FTLD-tau is grouped based on the tau isoform predominating the morphology. Pick’s disease (PiD) is a 3R tauopathy (60 and 64 kDa bands). 4R tauopathies (64 and 68 kDa bands) is comprised of progressive supranuclear palsy (PSP), corticobasal degeneration (CBD), argyrophilic grain disease (AGD), and globular glial tauopathy (GGT) [172]. Mixed 3R and 4R tauopathy (60, 64 and 68 kDa bands) is the neurofibrillary tangle (NFT)-dementia (discussed also in the frame of primary age-related tauopathy, PART), and this type of tau pathology is seen in Alzheimer diseased (AD) brains.

**Fig. 2:**
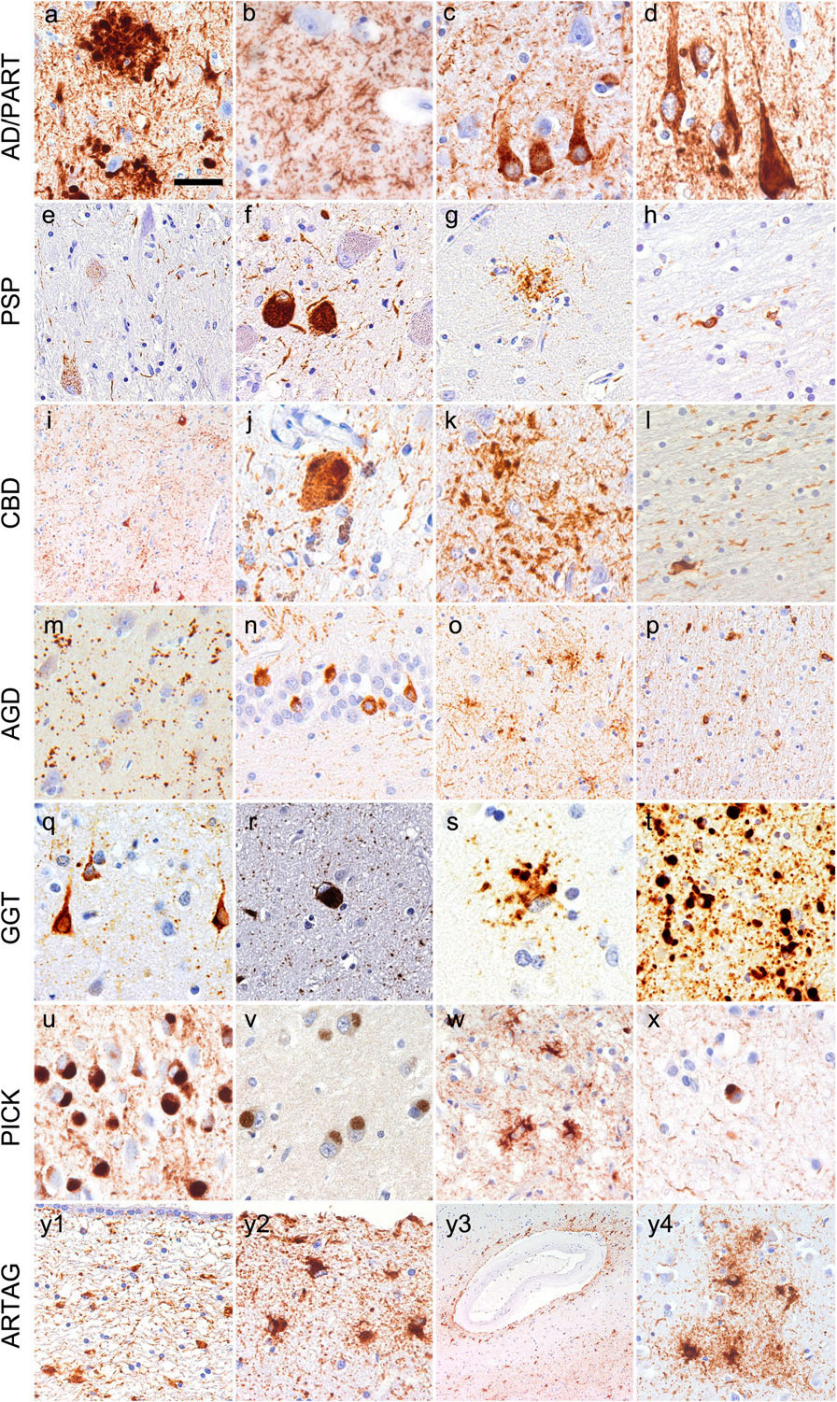
Tau pathologies in diverse tauopathies. Tau pathology in AD and PART comprise dystrophic neurites (*a*), axonal threads (*b*), pretangles (*c*) and NFTs (*d*). PSP is characterized by pretangles and threads (*e*), subcortical tangles (*f*), tufted astrocytes (*g*), and oligodendroglial coiled bodies (*h*). In CBD cases pretangles and threads (*i*), globose neuronal CBD-bodies (*j*), astrocytic plaques (*k*), and oligodendroglial coiled bodies (*l*) can be seen. AGD is characterized by 4R-tau positive neuronal dendritic grains (*m*), pretangles (*n*), granular/fuzzy astrocytes (*o*), and oligodendroglial coiled bodies (*p*). In GGT cases neuronal pretangles (*q*), spherical cytoplasmic inclusions (*r*), globular astroglial (*s*) and oligodendroglial (*t*) inclusions are detected. In Pick’s disease neuronal Pick bodies are frequent in the dentate gyrus (*u*) and show 3R immunoreactivity (*v*; here CA1 subregion is shown), furthermore, ramified astrocytes (*w*) and small globular oligodendroglial inclusions (*x*) can be noticed as well. Finally ARTAG comprises thorn shaped astrocytes and granular fuzzy astrocytes here demonstrated in the subependymal (*y1*), subpial (*y2*), perivascular (upper part of image 4) and white matter (lower part of image) (*y3*), and grey matter (*y4*) areas. All images show immunostaining for the AT8 antibody except (m) and (v) where immunostaining for 4R- and 3R-tau isoform, respectively, was performed. The bar in (a) represents 50 μm for a, e, f, g, h, l, m, t, u, v, y1, and y4; 35 μm for b, c, d, j, k, o, p, x; 30 μm for q and r; 40 μm for w and y2; 100 μm for i; 25 μm for s; and 150 μm for y3

Hyperphosphorylated tau is the major constituent of neuronal and glial inclusions, although there are further biochemical modifications (N- and C-terminal truncation, glycosylation, glycation, nitration of tyrosine residues, transglutamination, deamidation; acetylation; oligomer forms) [173] which are not examined routinely in diagnostic practice. Using phospho-dependent tau antibodies several morphologies of cellular tau immunoreactivity can be detected [172]. Tau immunoreactivity in neurons comprises pre-tangles (diffuse cytoplasmic neuronal tau immunoreactivity), NFTs, Pick bodies (3R-tau immunoreactive), spherical inclusions (usually 4R immunoreactive), dystrophic neurites, neuropil threads (axonal), and grains (dendritic). Astrocytic tau pathology includes tufted astrocytes (PSP), astrocytic plaques (CBD), ramified astrocytes (PiD), globular astroglial inclusions (GGT), thorn-shaped astrocytes, and granular-fuzzy astrocytes (the latter two seen mostly in age-related tau astrogliopathy, ARTAG). In oligodendrocytes, coiled bodies (PSP, CBD, AGD) and globular inclusions (PiD, GGT) can be detected (Fig. 2). The constellation of these morphologies and their anatomical distribution characterize primary tauopathies, e.g. NFTs in the medial temporal lobe is characteristic for PART [68] and NFTs in subcortical structures together with tufted astrocytes are pathognomonic for PSP [172]. Neuropathologic hallmarks of CBD comprise neuronal inclusions, threads in the white and grey matter, coiled bodies and astrocytic plaques [85]. AGD is characterized by the presence of argyrophilic and 4R tau immunoreactive grains in medial temporal lobe structures together with pre-tangles, oligodendroglial coiled bodies, and astrocytic tau pathology [324]. Globular oligodendroglial and astroglial inclusions characterize the GGTs [7]. PiD is a 3R tauopathy with Pick bodies, with less glial tau pathology and prominent FTLD [172]. In addition, neuronal tau pathology in the form of NFTs, threads and dystrophic neurites associated with Aß plaques is a hallmark of AD [39, 46, 91]. Finally, hereditary frontotemporal dementia (FTD) associated with mutations in the MAPT gene shows 3R-, 4R- and 3R/4R-tau pathologies overlapping with the neuropathologic features of primary tauopathies [101, 111]. However, in hereditary FTD, tau mutations lead to conformational changes before tau hyperphosphorylation [90].

Tau pathologies show hierarchical involvement of anatomical regions. This is exemplified by the six stages of NFT pathology in AD [38] and PART (usually only up to stage IV) [68] and the three stages of AGD-associated pathology [277]. For PSP and CBD hierarchical involvement is being studied; this is hindered by the heterogeneity of these diseases. A recent study described sequential distribution patterns of astroglial tau pathologies in CBD, PSP and in ARTAG types [175]. These observations on various stages complement experimental observations in cell culture and animal models, suggesting spreading of tau pathologies along neuronal connections and provide a basis for the concept of tau-strains as a background for disease heterogeneity [31, 60, 236, 280]. In fact, 3R, 4R and mutated tau species are likely to display different spreading behaviors [90]. Recent studies suggest that astrocytes might play a previously underappreciated role in the disease process. Indeed, astroglial tau pathology may precede neuronal tau immunoreactivities in primary FTLD-tauopathies [174, 193]. Astroglial tau pathologies might reflect their contribution to disease spreading or clearance of disease-associated proteins, and might lead to astroglial dysfunction contributing to neuronal degeneration [174].

## Pet imaging of tau pathology

Recently, the development of positron emission tomography (PET) radioligands presumably binding to tau has enabled the in vivo mapping and quantification of tau pathology, hitherto largely confirming autopsy findings. The radioligand [18F] Flortaucipir (FTP, previously AV1451 or T807), a benzimidazole pyrimidine derivative, is by far the most widely employed to date. It has been shown to bind with high affinity to mixed 3R- and 4R-tau isoforms in paired-helical filaments (PHF) of AD patients [26, 309, 361]. A recent study furthermore showed that in vivo FTP-binding and post mortem PHF load were highly correlated in a subject with a *MAPT* R406W mutation, which causes AD-like 3R/4R tau pathology [309]. However, large inter- and intra-individual differences were observed in a recent autopsy study of several tauopathies [361], calling for further investigation of FTP binding characteristics.

Off-target binding of tau PET ligands is another major limitation and challenge to be addressed in novel tracer development [26, 187, 200]. For example, the alleged tau PET ligand [18F]THK5351 demonstrated strong binding to monoaminoxidase B (MAO-B) *in* and ex vivo [133, 239], with ligand uptake being reduced by up to 50% in selected brain regions by the MAO-B inhibitor selegiline, preventing accurate quantification of tau [239]. Among the currently available tracers, the binding characteristics of FTP have been characterized best. FTP off-target binding has been observed in the caudate, putamen, and pallidum in elderly individuals regardless of their clinical diagnosis [20, 42, 205, 333, 354], and has been attributed to, amongst others, iron binding [59]. Its pronounced binding to the substantia nigra, also in cases with no apparent tau pathology, has been related to neuromelanin [219–221], as has elevated FTP binding in the pituitary gland, retinal pigment epithelial cells, leptomeninges, and malignant melanocytes in metastatic melanoma [205, 219, 221]. High FTP signal in the choroid plexus has been attributed to calcification/mineralization [205], binding to tangle-like structures corresponding to so-called Biondi ring tangles [150], or melanocyte binding [180, 219, 221] and constitutes an issue for the quantification of hippocampal ligand uptake due to their close proximity. Here, partial volume correction (PVC) might reduce bias from choroid plexus signal on hippocampal signal [180, 211, 212, 288]. FTP has also been shown to bind to MAO-A and B in vitro [335], however, no significant differences were observed in vivo between FTP scans of patients with and without MAO-B inhibitors [133].

A second generation of tau radioligands is supposed to be affected less by off-target binding issues, however, in vivo data are thus far limited for these ligands, which include, amongst others, [18F]RO6958948 (Roche) [142, 359], [18F]MK-6240 (Merck/Cerveau) [24, 199, 255], [18F]GTP-1 (Genentech) [278, 279, 350], [18F]PI2620 (Life Molecular Imaging, formerly Piramal Imaging) [314] and [18F]PM-PBB3 [249, 299].

For [18F] FTP, tracer uptake in physiological aging and AD appears to follow a particular spatial and temporal pattern. Although longitudinal data are limited to this date [153, 311], the distribution appears to begin in the entorhinal cortex, to spread into inferolateral temporal lobes and medial parietal lobes, and to eventually cover most of the neocortex in disease cases. To capture this high regionality, which is significantly different from e.g. PET imaging of Aβ pathology (often found throughout the neocortex), several approaches have been suggested for A) binary categorization of tau “positivity” [154, 212, 229, 344], and B) topographical staging approaches that recapitulate post mortem findings of tau distribution [211, 288, 290]. This regionality of tau PET ligand uptake in the brain is further emphasized by studies employing data-driven approaches without prior definition of anatomical regions [293, 352]. However, a few studies have suggested that ligand uptake assessment based on larger composite regions may be sufficient to capture AD-related tau PET signal and the longitudinal accumulation of tau [153, 211]. On a group level, FTP demonstrated clinical usefulness when its discriminative accuracy between AD dementia and non-AD neurodegenerative disorders was examined in a large multisite study, yielding very high sensitivity and specificity based on medial-basal and lateral temporal cortex ligand uptake [250].

In general, elevated tau tracer binding in the medial temporal lobe (MTL) can be observed in cognitively healthy older adults, whereas widespread binding in neocortical regions of any individual commonly is associated with the presence of cortical Aβ [58, 124, 161, 198, 211, 262, 288, 291, 294]. However, despite an overall correlation between brain Aβ and tau [161], the spatial distributions of these two aggregated proteins are discordant [161, 198, 294]. Interestingly, the strongest association can be observed between global Aβ and entorhinal tau PET signal [333], rendering this region important for the detection of AD-related tau PET signal.

Tau deposition outside the MTL is more common in individuals with AD; however, elevated tau tracer uptake has been reported for in neocortical areas in cognitively normal and even Aβ negative individuals [204]. While AD patients commonly have more widespread and pronounced tracer uptake than controls, exceptions have been found in AD patients who are Aβ-positive and show relatively low levels of tau deposition [262, 344]. Longitudinal studies have also demonstrated that increasing levels of Aβ are associated with more tau deposition in limbic and neocortical Braak regions several years later, even in nominally Aβ-negative individuals [179, 325]. Despite the limited availability of longitudinal data, it appears that tau accumulates over time in the temporal lobes of cognitively healthy individuals and AD patients, albeit this seems to be limited to Aβ-positive individuals [153, 311].

Compared to associations with Aβ, correlations between tau PET measures and age across healthy elderly seem to be weaker and confined to MTL regions [212, 289]. Greatest differences in FTP uptake between healthy young and elderly subjects are commonly observed in the choroid plexus and basal ganglia; however, tracer uptake in these regions most likely represents off-target binding [205, 206]. The age of symptom onset among AD patients clearly affects tau PET uptake patterns. Sporadic early-onset AD patients (EOAD) exhibit distinctly greater parietotemporal and frontal ligand uptake when compared with late onset AD (LOAD) which exhibits rather confined temporal lobe uptake [289]. Data from studies in early-onset familial/autosomal-dominant AD are limited, suggesting earliest FTP uptake in the medial temporal lobes of Aβ-positive presymptomatic mutation carriers but high cortical uptake, spatially comparable to sporadic EOAD cases in later symptomatic stages [268, 289].

Tau has, in contrast to Aβ, long been known to be much stronger associated with measures of cognitive decline and neurodegeneration [86, 88, 136, 155, 237]. In fact, greater FTP uptake has been shown to be related to both poorer cognitive function cross-sectionally and retrospective longitudinal decline in cognition functioning [13, 212]. In cognitively healthy elderly, associations are strongest between episodic memory performance and MTL, namely entorhinal cortical tracer uptake, whereas associations with global cognition are either absent or found for wider, less specific neocortical regions. Interestingly, the effect of MTL tau on episodic memory seems to be independent of global Aβ load [211, 288] both in these individuals and in individuals experiencing subjective cognitive decline [45]. Moreover, MTL tau accumulation in cognitively normal elderly is associated with patterns of neurodegeneration as assessed by both structural magnetic resonance imaging (MRI) and [18F] Fluorodeoxyglucose (FDG) PET that are topographically similar to the patterns seen in AD patients [2, 74, 125, 132, 176], suggesting that early-stage MTL tau might have a pathogenic role even in cognitively healthy individuals.

The relationship between tau, cognition, and neurodegeneration is even more pronounced in AD patients, especially in cases of EOAD who frequently exhibit language, visuospatial, or executive dysfunction rather than memory impairment and where the spatial distribution of tau deposition strongly reflects the clinical phenotype [250, 368]. In these patients, tau deposition is also strongly associated with the neurodegeneration markers of atrophy and glucose hypometabolism [27, 148, 250, 344], a relationship that cannot be explained by measures of or the distribution of Aβ [269]. Statistically, cognitive impairment can be related to both brain atrophy and tau, however, tau remains solely correlated with cognitive dysfunction, even when controlling for atrophy [23].

Generally, FTP uptake might be helpful in distinguishing clinical variants of AD, e.g. a recent study employing a data-driven clustering approach demonstrated that the majority of patients with relatively low entorhinal FTP uptake, compared to overall neocortical uptake, have an atypical clinical EOAD presentations, while most patients with high FTP uptake in both entorhinal and neocortex present with EOAD and a typical amnestic phenotype, and most with low FTP uptake in both entorhinal and neocortex present with typical LOAD [352].

In summary, the assessment of tau accumulation with PET has revealed a pattern of aggregation on a continuum from normal aging through AD that parallels neuropathological data and now offers the possibility of longitudinal studies. The strong relationship between tau PET measures and measures of neurodegeneration and cognition, taking in account the relationship between tau and Aβ, will elucidate how Aβ and tau pathology interact in the development of the processes that are linked to cognitive decline and clinical dementia.

## Extracellular and intracellular tau – Two sides of one coin

In pathological conditions, tau protein undergoes post-translational modifications, such as, truncation [241, 242, 357, 358], phosphorylation [127], ubiquitination [32, 181], glycation [283, 373], glycosylation [196, 343], nitration [144, 271, 272] and sumoylation [87, 209]. Among them, phosphorylation and truncation are the most studied. Many laboratories suggest that tau hyperphosphorylation on Ser and Thr residues facilitates tau aggregation. Tau is posttranslationally modified at Ser/Thr residies by O-linked N-acetylglucosamine (O-GlcNAc), and thus increasing tau O-GlcNAcylation may protect against tau aggregation. In tau transgenic mouse models, inhibition of β-N-acetyl-glucosaminidase, the enzyme responsible for O-GlcNAc removal, is protective [33].

It has been shown that truncated tau proteins are contained in the core of the paired helical filaments. Expression of the tau protein in the brain of transgenic rats and mice induced the formation of extensive neurofibrillary pathology, suggesting that truncated tau is a driving force of neurofibrillary degeneration [98, 381, 382, 384, 385].

Therapeutic approaches against tau pathology target either intracellular or extracellular tau or eventually both. It has been demonstrated that an increase in the level of intracellular tau could result in tau secretion into the extracellular space or in cell death [122, 304]. Toxic extracellular tau could interact with neuronal cell receptors such as M1/M3 muscarinic receptors [122, 123], or with heparin sulfate linked to cell membrane [372]. The result of that interaction could be again the onset of neuron toxicity and intracellular tau secretion. In this way, tau pathology could be propagated. Thus, possible therapies involving the use of muscarinic antagonists [131, 334, 336], or agents decreasing heparin sulphation [372], are under discussion for AD therapy.

Extracellular tau is found at significant levels in the interstitial fluid of the central nervous system (CNS), and can pass into cerebrospinal fluid (CSF) [370]. Initially, extracellular tau was thought to be only passively released by dying neurons, with selective vulnerability of neuronal types and cellular signals contributing to the disease progression [285]. However, there is now growing evidence that tau is actively transferred between neurons under pathological and physiological conditions. Aggregated and soluble tau variants have been shown to transfer between anatomically connected regions of the brain [75, 149, 197], and trans-synaptically between cells in culture [280, 363]. How tau is actively transferred between neurons is a major focus of dementia research, as attenuating the pathological spread may limit the progression of disease. Active tau transfer is thought to involve discrete steps including post translational modification (PTM), extracellular release and subsequent tau internalization.

Intracellular tau undergoes various PTMs including phosphorylation and proteolytic cleavage. Levels of total and phosphorylated tau detected in the CSF are important biomarkers for dementia [28]. Several tau modifications are detected at proportionally higher levels in extracellular compared with intercellular fractions, implicating specific tau modifications in active neuronal export [248]. Higher levels of extracellular aberrantly hyperphosphorylated tau are detected in patients with dementia [79]. Hyperphosphorylated tau has a lower binding affinity to microtubules (MT) [192] and mislocalizes to somatic and dendritic cell compartments [106, 143, 323]; these factors may contribute to active export as dissociation from MTs would allow a greater opportunity for tau to interact with components that facilitate protein export. C-terminally truncated tau (lacking approximately the last 50 amino acids) is detected at proportionally higher levels in the CSF samples of healthy individuals and dementia patients [284], and in neurons in culture [43, 163]. These tau species may be more readily detectable or resistant to degradation. Post-translational modifications and exon splicing events influence intra- and extracellular tau stability. Phosphorylated and 4R-tau isoform peptides have faster turnover rates than unphosphorylated and 3R-tau isoform peptides, respectively. Peptides from the N-terminal to mid-domain tau are more stable and have similar half-lives both inside and outside of the cell [284]. Notwithstanding these differences in stability, the proportionally higher levels of extracellular truncated tau suggest that physiological active tau release may be regulated by proteolytic cleavage.

Distinguishing between active tau release mechanisms and passive tau release, due to cell death, is challenging. The process of active tau release has been linked with several cellular mechanisms. In cell culture, monomeric tau can directly interact with the plasma membrane and proteoglycans, leading to unconventional secretion of tau [55, 164, 304], or the release of ectosomes containing tau [89]. Active tau release is also proposed to be regulated by neuronal activity. Depolarization of neurons promotes tau release [102, 263, 371] and release of exosomes containing hypophosphorylated tau [346]. These mechanisms aren’t mutually exclusive. However, it is unclear how they are associated and whether they relate to all forms of tau.

Following release into extracellular space, pathogenic tau can be taken up by healthy neurons, and promote seeded aggregation [165]. There have been conflicting reports regarding the forms of tau and route of entry of tau into various cell types. Studies suggest that aggregated tau is the predominant form internalized into cells [105, 362]. However, monomeric full-length tau can also be efficiently taken up by neurons [95]. These reports show that tau is taken up by endocytosis. Levels of the clathrin-mediated endocytosis component myc box-dependent-interacting protein 1 (BIN1) negatively correlate with tau uptake [52]. Different forms of tau enter neurons via distinct but overlapping pathways. Monomeric tau can enter neurons via dynamin-dependent endocytosis that is saturable, suggesting uptake is dependent on carrier proteins or receptors [95]. Entry of aggregated tau is attenuated by heparin in cell culture, indicating that heparan sulphate proteoglycans serve as receptor for tau uptake [140].

Hyperphosphorylated tau isolated from AD brain tissue is also recognized by the CNS immune system; microglia internalize and degrade tau in an Fc-dependent manner [210], and the cytosolic Fc receptor – tripartite motif-containing protein 21 (TRIM21), inhibits seeded tau aggregation [223]. Conversely, it is also suggested that the migration of microglia through the CNS transfers pathogenic species of tau to new areas of the brain [216].

It is currently unknown if tau transfer is a disease-specific phenomenon or physiological process appropriated during disease. Physiological tau transfer may be involved in network signaling or neuronal maintenance. Independently of the ability of pathological tau to seed aggregation, extracellular tau itself has been shown to be neurotoxic [84] and extracellular tau from individuals harboring amyloid precursor protein (APP) gene duplication can also cause synaptic dysfunction [145]. Tau immunotherapies that attenuate transfer of tau with the aim of limiting disease progression are under development [43, 64]. Tau antibodies have been shown to attenuate intracellular tau aggregation [375], while tau-antibody complexes can be internalized and targeted for degradation [56, 129, 215]. Identifying epitopes and conformations that distinguish between physiological and pathological tau transfer are important considerations when developing immunotherapies that target extracellular tau.

## Tau passive immunotherapy

In Alzheimer’s disease, tau protein is burdened by numerous post-translational modifications resulting in aggregation and tangle formation. Therefore, a number of passive vaccines for tau immunotherapy raised against various epitopes or conformation/s of tau have been developed, showing varied degrees of efficacy in attenuating tau pathology in animals, along with improvement in cognitive or motor functions. Several animal models have been used for testing of the therapeutic efficacy of monoclonal antibodies. Tau pathology is localized in various brain areas including hippocampus and brainstem. The location of tau pathology is mostly determined by the gene promotor. The clinical presentation is driven by topographic distribution of tau pathology, some of rodent models demonstrated cognitive decline while others suffer from impairment of sensori-motor functions [383]. The majority of preclinical studies have been performed on transgenic mice expressing mutant tau proteins (Table 1). However, tau mutations are not linked to familial forms of AD, but can cause frontotemporal dementia.

**Table 1:** Tau antibodies tested in preclinical efficacy studies

ANTIBODY	EPITOPE	ANIMAL MODEL	IMPROVEMENT		EFFICACY		REFERENCE
			Cognitive	Motor	NFTs	Insoluble tau	
PHF1	pS396/404	P301L	nd.	nd.	nd.	Reduced	[56]
		P301S	nd.	Improved	Reduced	Reduced	
MC1	aa7–9 and aa 313–322	P301L	nd.	nd.	nd.	Reduced	
		P301S	nd.	Improved	Reduced	Reduced	
MC1	aa7–9 and aa 313–322	P301L	nd.	nd.	Reduced	No change	[72]
DA31	aa150–190				No change	No change	
PHF1	pSer396/404				Reduced	Reduced	
4E6G7	379-408 (pS396/404)	P301L	nd.	nd.	Reduced	No change	[129]
6B2G12							
TOMA	nd.	Tg2576	Improved	Improved	nd.	Reduced	[54]
PHF6	pT231	rTg4510	Improved	No change	nd.	No change	[281]
PHF13	pS396	rTg4510	Improved	No change	nd.	No change	
		PS19	Improved	nd.	Reduced	No change	
HJ9.3	aa306–320	P301S	Improved	No change	Reduced	Reduced	[375]
HJ9.4	aa7–13		Moderate change	No change	Reduced	No change	
HJ8.5	aa25–30		Moderate change	No change	Reduced	Reduced	
HJ8.5	aa25–30	P301S	nd.	Improved	Reduced	Reduced	[374]
43D	aa6–18	3xTg-AD	Improved	nd.	Reduced	nd.	[73]
77E9	aa184–195		Improved	nd.	Reduced	nd.	
AT8	pS202 + pT205	3xTg-AD	nd.	nd.	Reduced	nd.	[342]
MAb86	pS422	TauPS2APP	nd.	nd.	Reduced	nd.	[61]
pS404 mAb IgG2	pS404	K3 and pR5	nd.	nd.	Reduced	Reduced	[151]
pS409-tau	pS409	P301L	nd.	nd.	Reduced	Reduced	[182]
Armanezumab	aa2–18	THY-Tau22	nd.	nd.	Reduced	nd.	[4]
PHF1	pS396/404	P301L	nd.	Improved	Reduced	No change	[36]
Ta9	pS396	tau609	Improved	nd.	Reduced	Reduced	[328]
		tau784					
Ta4	pSer396	tau609	Improved	No change	Reduced	Reduced	
		tau784					
Ta1505	pSer413	tau609	Improved	nd.	Reduced	Reduced	
DC8E8	aa268-273, aa299-304, aa330-335, aa362-367	R3/m4	nd.	nd.	Reduced	Reduced	[168]

*nd* Not defined

In general, tau therapeutic antibodies target, neutralize and/or eliminate either monomeric [36, 374, 375], aggregated forms [54], phospho-specific, or conformationally altered forms of tau protein [36, 56, 72, 129, 167, 342] (Table 1) and thus preventing formation neurofibrillary lesions. Anti-tau antibodies also differ in their binding site on tau. They recognise either the N-terminus [4, 73, 374, 375], the proline rich region [73, 342], the microtubule binding region [167, 375] or C-terminus [36, 56, 151].

The N-terminus of the tau protein has become attractive for preclinical development of tau therapeutic antibodies [4, 73, 374, 375]. This can be attributed to following reasons. Firstly, the conformational changes in the N-terminal region of tau occur very early in the disease pathogenesis in AD, which affects the function of the protein [62]. Furthermore, the exposure of the N-terminal is associated with early pathological event in human tauopathies [63]. The N-terminal fragment containing Gln124 displayed stronger ability to stabilize microtubules [78]. In addition, only N-terminal fragments were detected in the CSF from AD subjects [160, 284]. Similar results were also obtained from cortical neurons cultured from AD brains [43]. Moreover, the N-terminal fragment of tau protein was shown to increase amyloid beta production [43], and impair mitochondrial function, synaptic plasticity, and in turn was detrimental to neurons [9, 10, 34, 100]. Several studies focusing on antibodies targeting N-terminal sequences of tau have reported varied degree yet promising efficacy in reducing tau pathology and improveing cognitive or motor deficits during preclinical trials [4, 14, 73, 374, 375].

On the other hand, it has been shown that the majority of tau in the AD brain is truncated, mostly at the N-terminus [384]. A recent study showed that high molecular weight tau species from AD brain extract demonstrated strong immuno-positivity to C-terminal specific antibodies, and were weakly stained with N-terminal specific antibodies, indicating substantial lack of N-terminal sequences in oligomers and fibrils from the AD brain [380]. In concordance with this study, two recent papers demonstrated that N-terminal tau antibodies do not recognise truncated tau and the whole spectrum of aggregated forms of tau in Alzheimer’s disease brain. They mainly decorate a triplet of hyperphosporylated full-length tau – A68 [183]. This means that a large portion of pathological tau is not recognised by N-terminal tau antibodies [67, 331, 380]. By using a seeded aggregation cell model, N-terminal antibodies (PT26, aa 23-26; PT93, aa27-32; hTau10, aa29-36) showed incomplete depletion of human-derived seeds even at the concentration, which was sufficient for complete depletion of tau seeds from P301S transgenic model (300 nM) [331]. Similarly, two tested N-terminal antibodies (aa15-24, aa 25-30) and MC1 (which recognises both N-terminus and microtubule binding domain) failed to fully prevent seeding of AD tau in a seeded aggregation cell model [67] and in vivo [8]. In contrast, Nobuhara and colleagues [240] demonstrated that N-terminal antibody C13 (aa2-18) efficiently removed tau from rTg4510 brain extracts and human AD high molecular weight tau (HMW). Moreover, the antibody reduced tau uptake of pathological mouse and human AD HMW tau in a sensitive FRET-based in mouse primary neurons. It is important to note that the antibodies targeting the N-terminus on tau are not specific to diseased tau, and they possibly reduce the level of physiological tau.

While beneficial effects of N-terminal antibodies on reduction of tau uptake or inhibition of seeding activity are still a matter of discussion, the development of novel therapeutic tau antibodies has shifted to the mid domain of tau protein. In the mid region, phosphorylation of tau at the position pS202 and pT205 was reported as an intracellular and extracellular marker for tau pathology in AD [39], and is potentially involved in neuronal apoptosis [166]. Moreover, phosphorylation of tau at T231 was also reported as an early event in AD [207, 208]. Several mid-domain tau antibodies (PT51, aa153-158, PT79, aa131-140, PT89, aa173-178) demonstrated complete depletion of mouse transgenic tau P301S-derived tau seeds. However, incomplete depletion of human derived seeds even at maximal concentration of 300 nM [331], suggests the different composition of mouse and human tau seeds. On the other hand, the antibody 6C5 (aa125-131) efficiently removed tau (> 85% reduction) from both mouse transgenic (Tg4510) brain extracts and human AD HMW tau (82% reduction). Furthermore, the antibody was the most effective in reducing tau uptake of pathological mouse tau (> 90% reduction) and human AD HMW tau (> 75% reduction) as well in a sensitive FRET-based assay in mouse primary neurons [240]. Similarly, the antibody recognising aa235-250, fully neutralised seeding activity of AD and PSP tau in a seeded aggregation cell model with an IC50 of 2.9 nM and 5.6 nM, respectively [67]. These results demonstrate that antibodies recognising the mid region of tau can be effective in the reduction of tau uptake and neutralisation of tau seeding activity. In contrast to in vitro experiments, studies using tau antibodies raised against this region of tau showed inconsistent results in preclinical in vivo experiments [72, 73, 342].

The third class of antibodies target the microtubule binding region (MTBR), which plays a crucial role in polymerization and stability of microtubules [36, 168, 328]. On the other hand, this region is responsible for the pathological tau-tau interaction. It was reported that the C-terminal fragments were more prone to filament formation than the N-terminal sequences [257, 258]. Specifically, the region spanning aa244-372 corresponds to the amyloid-forming region on tau protein [315]. This property is attributed to the hexapeptide sequence _306_VQIVYK_311_ on the 2nd repeat of MTBR which was shown to promote tau aggregation by a nucleation dependent mechanism [338]. Recent cryo-electron microscopy study demonstrated that this hexapeptide packed through a heterotypic, non-staggered interface with the opposing residues 373–378 [99]. Moreover, the hexapeptide on the 3rd MTBR also caused formation of fibrils in vitro [315]. Currently, only two preclinical studies on passive immunotherapies targeting the MTBR were performed, both showing promising results [168, 375]. More specifically, the antibody DC8E8 [168] binds to the four highly homologous and yet independent hexapeptides localised in each microtubule binding domain, while mAb HJ9.3 (epitope 306-321) recognises the hexapeptide sequence _306_VQIVYK_311_ [375]. Both antibodies were effective in reduction of neurofibrillary pathology in the brain of transgenic rodent models.

It has been shown that the C-terminus enhanced the microtubule binding capacity of tau protein and also influenced pathological tau aggregation [177, 232]. More specifically, the C-terminal region of tau harbors several phosphorylation sites which regulate microtubule binding of tau and hyperphosphorylation of phospho-sites in this region, such as pS413, pS396, pS404, are observed in early and late stages of AD progression [15, 300]. Therefore, several studies are devoted to investigating the effect of C-terminal specific tau antibodies in animal models [36, 56, 129, 151, 182, 328].

Finally, conformational changes and oligomer formation of tau protein represent early events in the pathogenesis of tau lesions in AD [39, 256, 348]. For example, with MC1 (aa7–9 and aa313–322), conformational epitope specific reactivity is observed in Braak stages I and II in AD [348]. In addition, MC1 immuno-purified soluble tau species readily assembled into paired helical filaments in vitro [348]. Therefore, antibodies against these unique species of tau are also being investigated in preclinical studies to attenuate tau pathogenesis. MC1 therapy slightly reduced insoluble tau and number of tangles in the brain of experimental mice [54, 56, 72].

Currently, only a handful of humanised tau antibodies are being investigated at various stages of clinical development (Clinicaltrials.gov). Humanized versions of N-terminal specific antibodies 8E12 [374, 375], and BIIB092 (also known as BMS-986168 or IPN007) [43] are being currently investigated at various phases of trials for treatment of PSP and AD. Another N-terminal antibody RO 7105705 (RG 6100) has already entered Phase 2 clinical trials, targeting Alzheimer’s disease. Janssen is also starting phase 1 clinical trials in mild AD with the antibody JNJ-63733657 which is effective at eliminating pathological tau seeds. Antibody UCB0107 that targets the mid region of tau is currently in Phase I (healthy volunteers). Antibody LY3303560 (modified MC1 antibody) recognising both N-terminus and microtubule binding domain is in Phase 2 trial in MCI-to-AD or mild to moderate AD patients. Finally, antibody BIIB076 that has the ability to bind monomeric and fibrillar forms of tau is being tested in the Phase I clinical trial in AD [65, 71](www.alzforum.org).

There are several advantages of passive immunotherapy. In terms of pharmacology definition, antibodies are precisely characterised both in vitro and in vivo (avidity, affinity, target specificity, half-life, concentration, single isotype). Passive immunotherapy does not require the immune system to generate an immune response. The main disadvantages are expensive production, the short half-life of antibodies and chronic systemic administration (i.v.). Chronic administration may lead to formation of anti-antibodies, which could result in neutralization and/or have other unwanted immunological side effects [128].

### Importance of binding mechanism and affinity of therapeutic anti-tau antibodies

The binding of antigen by an antibody is effectuated by direct contacts between the antigen epitope and antibody complementarity determining regions (CDRs). The three-dimensional structure of the CDRs and its temporal fluctuations conditioned by the flexibility of the antibody molecule determine (1) the *specificity* for an epitope, (2) the binding *selectivity* between various presentations of the epitope and (3) the *strength* of interaction (the stability of the antibody-antigen complex), where the strength is quantified as association (equilibrium) constant, K_a_, or its reciprocal quantity, the dissociation constant K_d_. All these aspects are interconnected, where the latter, quantified strength of interaction, is being used for the determination of previous two, i.e. specificity and selectivity.

According to the available data, not all three of the abovementioned aspects were evaluated for all the anti-tau therapeutic antibodies. The specific epitopes are the best characterized and thoroughly described in a recent review [189, 244]. They comprise linear, conformational or phosphorylation-dependent sites on tau [302]. The second aspect, selectivity towards pathogenic presentation of the epitope, is important for both the safety and the efficacy of the anti-tau therapy. This avoids the side effects caused by knocking out healthy tau and focuses the antibody action towards the initial and/or the most toxic pathological tau forms. In this respect, some of the antibodies have had claims for their selectivity for pathological tau at various stages of tau neurodegeneration, e.g., MC1 for a conformation associated with tau filaments [99, 159], ACI-5400 for a phospho-epitope inducing a pathological conformation [321] or DC8E8 for multiple epitopes selectively presented on conformational ensemble of pathogenic truncated tau [168, 243]. The third aspect, interaction strength, has been frequently evaluated by relative quantification on western blot, or, more precisely, by ELISA. For an absolute quantification the surface plasmon resonance (SPR) technique has been used.

A confusing aspect of quantification of binding strength arises in the distinction between monovalent and multivalent arrangement of the quantification protocol. A full-length monoclonal antibody of IgG class contains two binding sites for the antigen. For determination of binding strength, one has to measure K_A_ or K_D_ of interaction of *one* binding site with *one* epitope on the antigen molecule, e.g. using monovalent antibody Fab. This quantity is commonly called as antibody *affinity.* The affinity is a *constant value*, characteristic for the given antibody binding site – antigen epitope pair, and may be used for an unbiased comparison of antibody binding strength. Affinity is independent of the spatial arrangement of antigen. If performed properly, it is independent on the design of the measurement.

The strength of binding of a whole IgG molecule, which is bivalent, may be expressed equally as a K_A_ or K_D_, but with this we measure the *avidity* of antibody. The *avidity is not a constant* and depends on the availability of the antigenic epitopes in the vicinity of both IgG antibody binding sites *simultaneously*. When an epitope is present at a high local concentration (that is, at a high areal/spatial density), e.g. on a surface (during Western blotting, on the ELISA plate/SPR sensorchip with a high density of immobilized protein etc.) or on the polymerized antigen (tau filaments), the overall level of bound antibody may be very high with the probability that at least one of the antibody binding sites can at any one moment be bound to the antigen.

*A*ntibody avidity is effective in situ (in the inter-neuronal space) towards protein particles with a high spatial density of its epitopes (e.g. oligomerized, aggregated and filamentous tau, but not monomeric tau). Generally, the avidity of a mature, functional antibody can reach extreme values, ranging from 10^− 12^ to 10^− 15^ M (picomolar to femtomolar), whereas the affinity of a single antibody binding site is proportionally lower, in the range of 10^− 8^ to 10^− 10^ M (nanomolar to subnanomolar). It is of note that the immune system employs an affinity ceiling at ~ 10^− 10^ M during antibody maturation, eliminating the antibodies with excessively high affinities, that are not beneficial for the organism [22]. It was postulated that for therapeutic antibodies for tauopathies, a strong selectivity towards pathological tau may be more important than high affinity [72, 301].

Whereas affinity, the constant measure characteristic for a given antibody-antigen pair can be quantified reproducibly on different SPR instruments in different laboratories, using various immobilization chemistries and a range of time kinetic protocols, the avidities are more difficult to reproduce with a new sensorchip or with different arrangement of measurement, because they are intrinsically dependent on the conditions of measurements. It is known that a low flow rate used in SPR could artificially decrease the dissociation rate constant and therefore increase the affinity due to rebinding events [234]. Equally, the amount of protein on the chip could also increase rebinding and mass transport artefacts [235].

Reactivity of anti-tau antibodies HJ8.5, HJ9.4 and HJ9.3 were measured at conditions where the avidity was effective due to the use of bivalent full-length antibodies, and a very high density of tau epitopes on the surface of sensorchip [375]. Therefore, determined values represent avidity rather than affinity. Reactivity of antibody ACI-5400 was also measured with bivalent full-length antibody, but with a low density of epitopes on the sensorchip [321]. Therefore, the determined value likely corresponds to the affinity; although a correction for a bivalent analyte has to be performed. Antibody DC8E8 was measured with low densities of antibody on the sensorchip, therefore, strictly under conditions measuring affinity, and thus, the values represent affinities [167] (Table 2).

**Table 2:** Overview of affinity/avidity data of candidate therapeutic antibodies

ANTIBODY	EPITOPE	AFFINITY	AVIDITY	TARGET (IN SPR)	NOTE	REFERENCE
HJ8.5	aa25-30	nd.	0.4 pM	Human Tau2N4R	tau immobilized to a high level (> 3000 RU)	[375]
HJ9.4	aa7-13	nd.	7 nM	Human Tau2N4R	tau immobilized to a high level (> 3000 RU)	[375]
HJ9.3	aa306-320	nd.	100 pM	Human Tau2N4R	tau immobilized to a high level (> 3000 RU)	[375]
ACI-5400	aa393-408(pS396)	38 nM	nd.	Tau393-408(pS396/pS404)	tau immobilized to a low level (130 RU)	[321]
DC8E8	Tetratope in the repeat region of tau (aa268-367)	91 nM	nd.	Human Tau2N4R	Antibody immobilized to a low level (230-250 RU)	[168]
DC8E8	Tetratope in the repeat region of tau (aa268-367)	14 nM	nd.	Pathological Tau151-391_4R	Antibody immobilized to a low level (230-250 RU)	[168]
derived from MC1	aa7-9; aa312-322	235	nd.	monomeric tau	Antibody immobilized at unknown density	[135]
derived from MC1	aa7-9; aa312-322	nd.	< 0.22 nM	tau aggregate	Antibody immobilized at unknown density	[135]

*nd* Not defined, *SPR* Surface plasmon resonance spectroscopy

For unbiased comparison of binding strength and specificity of candidate therapeutic anti-tau antibodies, the affinity should be strictly used. Binding of therapeutic antibody to oligomerized tau protein species in the interstitial brain space would benefit from increased avidity of a bivalent antibody, assuming that the antibody epitope is present on the polymerized tau in sufficiently high spatial density. The latter requirement might be fulfilled for repeat region-directed antibodies, as the repeat region is the constitutive component of the core structure of assembled tau [99, 242]. The avidity enhancement for binding of N-terminal anti-tau antibodies like HJ9.4 and HJ8.5 is compromised from two reasons: (1) the N-terminal part of tau is not regularly arranged in the tau polymers, but rather forms a fuzzy coat [99] and (2) a significant portion of high molecular weight tau species in the Alzheimer’s brain is N-terminally truncated [384] and may be lacking the antibody epitopes.

## Tau therapeutic vaccines

Like their passive immunotherapy counterparts, active vaccines targeting the mid-region, microtubule binding domain and C-terminus have been extensively investigated in preclinical studies (Table 2). Most of these studies demonstrated reduction in tau pathology [14, 30, 167, 270, 274, 322] along with improvement in cognitive or sensorimotor abilities in animals [36, 37, 167, 322, 326] (Table 3).

**Table 3:** Preclinical studies on tau vaccines

IMMUNOGEN	ANIMAL MODEL	IMPROVEMENT		EFFICACY		REFERENCE
		Cognitive	Sensorimotor	NFT’s	Insoluble tau	
Tau379–408 [pS396,404]	P301L	No change	Improved	Decreased	Decreased	[14]
Tau 379-408 [pS396/404]	htau/PS1M146L	Improved	Improved	Reduced	Reduced	[36]
Tau 417-426 [pS422]	Thy-Tau22	Improved	nd.	Reduced	Reduced	[326]
Tau393-408 [pS396/S404] (Liposome based)	P301L	nd.	Improved	No change	Reduced	[322]
Tau379-408 [pS396/S404]	hTau X PS1	Improved	No change	Reduced	Reduced	[37]
	hTau	Improved	No change	No change	Reduced	
	hTau/PS1/mTau	Improved	No change	No change	Reduced	
Tau195-213 [pS202/T205]	DM-Tau-tg	nd.	nd.	Reduced	nd.	[30]
Tau207-220 [pT212/S214]		nd.	nd.	Reduced	nd.	
Tau 224-238 [pT231]		nd.	nd.	Reduced	nd.	
Tau aa395-406 [pS396/404]	pR5	nd.	nd.	Reduced	nd.	[25]
Human paired helical filaments (PHF’s)	THY-Tau22	nd.	nd.	Reduced	Reduced	[12]
Tau229-237 [pT231/pS235]	P301S	nd.	nd.	nd.	nd.	[273]
	Tg2576					
Tau199–208 [pS202/pT205]	P301S	nd.	Improved	No change	No change	[274]
		nd.	Improved	No change	No change	
Tau209–217		nd.	Improved	No change	No change	
Tau 294-305	SHR72 rats	nd.	Improved	Reduced	Reduced	[167]
Tau 379-408 [pS396/404]	3xTg-AD	No change	nd.	Reduced	Reduced	[270]
Tau 294-305	P301S	Improved		Reduced	Reduced	[157]

*nd* Not defined

Interestingly, the majority of preclinical studies with tau active vaccines have paid only marginal attention to the characterization of the antibody response induced by the vaccines. It should be emphasized, that the primary goal of all designed tau vaccines is antibody-mediated protection. The quantity and quality of the vaccine antibodies may represent a critical correlate of the efficacy of tau vaccines. In general, the measurement of titer or concentration by ELISA is the widely accepted approach for quantification of antibody response in body fluids [66, 369]. Unfortunately, there is still no agreement on the optimal methods for measurement of anti-tau antibodies, or how the results of such assays should be reported [3]. Many preclinical studies of the tau vaccines have analysed the antibody response in a rather descriptive manner as “good, robust, high or low”, and did not elaborate on its quantitative aspect [14, 37, 270, 322]. Only two studies published so far, have defined the titer of the antibody response [167, 274]. There is an urgent need for development of common standards for the measurement of antibody response with the most sensitive and reproducible methods. This will allow us to perform a direct comparison of antibody responses between different assays and different clinical trials [3]. Another determining factor of vaccine efficacy is quality of vaccine-induced antibodies (e.g., their isotypes, affinity/avidity, target epitope, functional activity). For example, antibody isotype already more or less indicates antibody affinity. Moreover, to some extent, affinity reflects therapeutic effectivity of antibody.

**Fig. 3:**
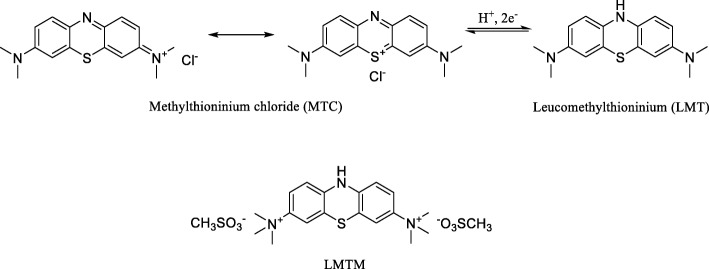
Chemical structures of methylene blue derivatives

In comparison with passive tau immunotherapy, there are only two tau active vaccines that have been tested in human clinical trials, AADvac1 for Alzheimer’s disease and non-fluent primary progressive aphasia (Axon Neuroscience SE), and ACI-35 vaccine for Alzheimer’s disease (AC Immune SA, Janssen). Active vaccine AADvac1 consists of tau peptide (aa 294-305/4R) that was coupled to keyhole limpet haemocyanin (KLH) in order to stimulate production of specific antibodies. The 24-week first-in-man study on AADvac1 in patients with mild to moderate AD dementia demonstrated encouraging results in both safety and immunogenicity. Twenty nine of 30 patients developed an IgG response against the tau peptide component of AADvac1 and against recombinant pathological tau (aa151-391/4R) [381]. The serum antibodies showed a pronounced preference for pathological truncated tau over healthy full-length tau protein [245]. Similarly, a 72-week open-label single arm interventional follow-up trial (FUNDAMANT) displayed a benign safety profile of the vaccine. No cases of meningoencephalitis or vasogenic oedema were observed. There was a tendency towards slower atrophy in MRI and less decline in cognitive assessment in patients with high titres [243]. Currently, a phase II clinical trial in AD and a phase I trial in non-fluent primary progressive aphasia are underway (alzforum.org) (Fig. 3).

Much less is known about the ACI35 clinical trial. ACI-35 is a liposome-based vaccine consisting of a synthetic peptide to mimic the phospho-epitope of tau at residues pS396/pS404 anchored into a lipid bilayer. A phase 1b multi-centre double-blind randomized placebo-controlled trial in 24 patients with mild to moderate Alzheimer’s disease compared low, medium, and high doses of the vaccine to placebo.

Active immunization is long lasting because it induces immunological memory. Active vaccines are easy to administer (different routes) and the production is cost-effective. Immunisation generates polyclonal response; antibodies can recognize multiple epitopes on the target protein with different affinity and avidity. On the other hand, the immune response depends on the host immune system, there is a variability in the antibody response across patients [128, 353].

## Antisense therapies for tauopathies

Direct targeting of tau gene (*MAPT*) expression is gaining currency as a therapeutic approach with an antisense oligonucleotide (ASO) therapy already in Phase I clinical trials. Several in vivo and cell studies have demonstrated the benefit of tau reduction in slowing pathological progression and improving functional deficits in tauopathy models both dependent and independent of ß-amyloid pathology. Tau reduction also results in significant improvements in seizures associated with AD pathology and in a model for Dravet syndrome [112].

The fibrillar tau pathology in tauopathy brains consists of abnormally hyperphosphorylated tau protein [169, 360]. Normal phosphorylation and dephosphorylation of residues within and flanking the microtubule (MT)-binding repeat domain (MTBR) mediates the dynamic binding and release of tau from MTs [303]. Hyperphosphorylation could cause or be the result of aberrant release of tau from MTs, with hyperphosphorylated tau unable to bind to MTs [41]. The resulting surplus of unbound tau together with localised concentrations, could lead to the triggering of pathological conformational conversion of tau to a seed-competent form [228] and the initiation of the aggregation cascade that leads to the fibrillar tau accumulations.

The genetics of tau have informed us on the role of tau defects as directly contributing to neurodegeneration. The early dominance of Aß and the amyloid hypothesis [292] subsumed tau to a consequence or bystander in the AD pathogenesis cascade. However, it was clear that the spread and severity of tau pathology better correlated with clinical progression of AD [40, 116, 126]. The identification of mutations in the tau gene (*MAPT*) that cause familial forms of FTLD with tau pathology (FTLD-tau) [147, 313] cemented the primary role of defective tau as a neurodegenerative agent. From these genetic studies, the identification of common genetic variation in *MAPT* emerged, defining the H1 haplotype, that is a strong risk factor for primary tauopathies with dominant 4R-tau pathology, progressive supranuclear palsy (PSP; OR = 5.46) [19, 139, 260] and corticobasal degeneration (CBD; OR = 3.7) [139, 147, 171] and, more surprisingly, Parkinson’s disease (OR = 0.77) [306].

The FTLD-tau mutations in *MAPT* fall into two broad classes; missense mutations that chiefly affect residues within the MTBR that impair microtubule binding capacity and/or increase fibrillogenicity of tau, and splicing mutations in intronic sequences flanking the alternatively spliced exon 10 and in splicing regulatory motifs within exon 10 [147]. The latter cause increased inclusion of exon 10 and ensuing increased ratio of tau isoforms with four MTBRs (4R-tau) over those containing three MTBRs (3R-tau) [118]. The splicing of *MAPT* exons 2, 3 and 10 is developmentally regulated, and in the healthy adult brain, there are about equal amounts of 3R- and 4R-tau [117, 170]. The basis of the increased risk conferred by the H1 haplotype of *MAPT* and its defining common polymorphisms, spanning the entire gene and beyond, could be the demonstrated allele-specific differences in transcription [233] and of splicing of exons 3 and 10 of the *MAPT* pre-mRNA [50, 233]. The result is an overall increase in tau levels, particularly the more fibrillogenic 4R-tau, leading to the 4R-tau dominated pathology seen in PSP and CBD [195]. Furthermore, it was shown that 17q21.31 duplication leads to early-onset dementia with an AD clinical phenotype [178].

## Therapeutic reduction of tau

Surplus availability of unbound tau, particularly of the more fibrillogenic mutants or 4R-tau could, with abnormal hyperphosphorylation, lead to mislocalisation and aberrant interaction with other cellular components and milieux. This leads to conformational conversion of tau from its highly soluble, intrinsically disordered characteristic to the seed-competent aggregation-prone form [228]. This has led to the notion that reduction of total tau (or surplus 4R-tau) could be therapeutically beneficial. Although the recent stable of passive immunotherapy approaches targeting tau could be blockading intercellular transmission of pathological tau seeds, a plausible mechanism could also be a reduction of pathological tau mediated by microglial or neuronal uptake and clearance of extracellular tau-antibody complexes [107, 210, 223].

Several published pre-clinical studies with cell and animal models of AD and tauopathies have persuasively demonstrated the possible therapeutic benefit of tau reduction (Table 4). An ASO-based approach already having entered Phase I of clinical trials [227]. In early work, SantaCruz and colleagues demonstrated recovery of memory function and reduced neuronal loss after conditional repression of tau expression in the rTg4510 mouse [282]. Reduction of endogenous tau levels in AD mouse models overexpressing human amyloid precursor protein (hAPP) with familial AD mutations dose-dependently ameliorated Aß-related learning and memory deficits and protected the mice from early mortality [152, 275]. The benefit of tau reduction occurred without influencing Aß burden suggesting that tau reduction uncouples Aß from downstream pathogenic mechanisms [275] including the prevention of Aß-induced defects in axonal transport [341]. Other mouse studies have also shown tau reduction-mediated mitigation of cognitive deficits as a consequence of mild repetitive brain injury [57], or type-1 diabetes [1].

**Table 4:** Studies on cell and animal models demonstrating therapeutic benefit of tau reduction

MODEL	BENEFITS	REFERENCES
Tet-repression of Tg-tau expression in rTg4510 mice	Reduced neuronal loss and improved memory function	[282]
hAPP tau−/− crosses	Blocks Aß and excitotoxin mediated neuronal dysfunction	[275]
hAPP (APP23) Dtau or tau−/− crosses	Prevention of Aß-mediated memory deficits and improved survival	[152]
CSF delivered ASOs	Reduces evoked seizures in adult nTg mice	[81]
tau−/− *Kcna−/−* crosses	Reduced network hyperexcitability in mouse and *Drosophila* epilepsy models	[141]
Crossing tau−/− mice with nTg mice	Reduces learning and memory deficits due to mild repetitive traumatic brain injury in mice	[57]
Streptozotocin-treated tau−/− and nTg mice	Mitigates cognitive deficits in type-1 diabetes mouse model	[1]
tau−/− *Scn1a −/−* R1407X loss-of-function truncation mice	Prevents seizure and improves survival in Dravet syndrome mouse model	[112]
shRNA knockdown of *Mapt* in nTg mouse primary neurons	Prevents Aß-induced axonal transport deficits	[341]
ASO knockdown of Tg-tau overexpression in PS19 mice	Reduced tau pathology, reversal of existing tau pathology. Prevention of neuronal loss. Improved behavioural deficits	[82]
Inducible tau knockdown in APP/PS1 x rTg4510 mice	Prevents tau pathology and neuronal death in presence of Aß pathology	[80]

Abbreviations: *Tg* transgenic, *nTg* non-transgenic (wild-type), *Tet* tetracycline, *hAPP* human amyloid precursor protein, *shRNA* short hairpin RNA

With excitotoxicity implicated in AD, and increased incidence of seizures in AD patients [11], tau reduction also prevented increased susceptibility of hAPP mice to evoked seizures [275]. This protection extended to seizures independent of AD pathology with ASO-mediated knockdown of endogenous tau in adult non-transgenic mice [81] and in mouse (*Kcna1*^−/−^) and *Drosophila* (*kcc* and *eas*) models of hyperexcitability [141] as well as a mouse model for Dravet syndrome [112].

## Antisense therapies

This is an exciting juncture in the hunt for therapies against neurodegenerative disorders by directly targeting those causative genes. The efficacy and safety of ASO therapy has been demonstrated in clinical trials for nusinersen (*Spinraza*®; ClinicalTrials.gov Identifier: NCT02193074) for the treatment of spinal muscular atrophy (SMA) and eteplirsen (*Exondys51*®; NCT00844597, NCT01396239/NCT01540409, NCT02255552) to treat Duchenne muscular dystrophy (DMD). More recently, IONIS-HTT_Rx_ (RG6042; NCT02519036) was tested for the treatment of Huntington’s disease (HD) [317]. This specifically targets the mutant, expanded huntingtin gene (*HTT*) mRNA and supresses its expression. A recent Phase 1/2a clinical trial with intrathecal delivery of the ASO has had no adverse drug-related incidents and showed promising reduction of mutant *HTT* mRNA levels in CSF [317].

ASOs are short, single-stranded oligonucleotides (8-50 nucleotides) that are designed to bind with complete specificity to complementary sense pre-messenger RNA (mRNA) or mature mRNA sequences. Depending on design and binding site, they could mediate degradation of the target mRNA or prevent translation and thus attenuate protein production. Gene down-regulation by ASOs exploits cellular mechanisms either via RNA interference (RNAi) and the degradation of the target mRNA by RNA-induced silencing complex (RISC), or by recruitment RNase H1 to degrade mRNA at the site of the DNA-RNA duplex. Owing to their size and highly charged nature, ASOs present challenges in terms of cellular uptake, stability and susceptibility to degradation by nucleases and, particularly with CNS targeted therapies, overcoming the blood-brain barrier (BBB). These can in part be overcome by chemical modifications of the DNA or RNA phosphodiester backbone or ribose sugar [190] and the use of the likes of viral vectors, liposomes, polyplexes, or cell-penetrating peptides to enhance delivery [96, 222, 367].

Based on the striking success and safety profile of recent ASO-based clinical trials and, and the recent in vivo ASO-based tau reduction work by de Vos and colleagues [80], a clinical trial of IONIS-MAPT_Rx_ (BIIB080, ISIS 814907), the first ASO targeting tau in mild AD patients, is currently under way [ClinicalTrials.gov Identifier: NCT03186989]. Via repeated intrathecal delivery, it appears that this ASO can overcome the BBB in non-human primates with about 75% reduction of *MAPT* mRNA in both hippocampus and cortex and no dose-limiting side-effects [227].

As shown with nusinersen in SMA and eteplirsen in DMD, ASOs could also be used to target splice acceptor or donor sites or splicing enhancers or repressors to block or enhance splicing of alternatively spliced exons [69, 190]. SMA is caused by survival motor neuron 1 (*SMN1*) gene mutation causing loss of SMN1 protein, resulting in loss of motor neuron function [202]. The intrathecally administered ASO targets the paralogous *SMN2* pre-mRNA, promoting inclusion of exon 7 and production of active SMN in place of the depleted *SMN1* product [307]. DMD is a fatal X-linked recessive neuromuscular disorder characterised by progressive muscle weakening and wasting caused by disruptive mutations throughout the large (79 exon) *DMD* gene [203]. ASO approaches for DMD, including eteplirsen, are designed to induce exon skipping, thereby excluding dispensable downstream exons and avoiding exons with disruptive loss-of-function frame-shift or splice site mutations, while still producing an internally truncated, partially functional protein [190].

Noting the pathogenic role of increased availability of 4R-tau due to exon 10 mutations in FTLD-tau and the *MAPT* H1 haplotype in PSP and CBD, rebalancing exon 10 is also being tested [276, 287]. This includes ASO-based targeting of exon 10 splice motifs leading to exon-skipping and reduced 4R-tau [287], or reprogramming using a spliceosome-mediated *trans-*splicing (SMaRT) technique that acts by creating a hybrid mRNA through a *trans-*splicing reaction between the *MAPT* pre-mRNA and a pre-*trans*-splicing molecule, comprised of a binding domain that hybridises with the 3′ end of intron 9 and exons 11–13, designed to exclude exon 10 [276].

### *MAPT-AS1* natural antisense transcript as a physiological repressor of tau expression

In addition to ASOs, we have seen recent upsurge in our understanding of natural antisense transcripts (NATs). These are endogenous RNA molecules formed by antisense transcription at coding genes and play multi-layered role(s) in regulation of expression of their paired coding gene [347]. The *MAPT-AS1* long non-coding RNA (lncRNA) gene partially overlaps head-to-head with the promoter and 5′ untranslated region (5’-UTR) of *MAPT* and by alternative splicing and use of alternate exons and splice sites, expresses multiple NATs (tau-NATs) [305]. Both in vitro and in vivo, some of the tau-NATs potently repress tau translation [305]. This presents a novel, physiological repressor of tau protein acting in the cytoplasm that, unlike synthetic ASOs, does not rely on RISC or RNAseH and is amenable to adeno-associated virus (AAV) vector-based delivery. Several clinical trials using AAV vectors, including intracranial delivery, have been shown to be safe [137]. Widespread CNS distribution and persistence for up to 10 years and no adverse effects [188] could imply treatment limited to a single delivery unlike ASOs where in ongoing clinical trials, involve repeat intrathecal injection of large doses, every few weeks over several months.

### Consequences of tau reduction

Given the importance of tau in multiple facets of neuronal function, mainly by its role in axonal MT assembly and stabilisation and mediation of axonal transport, deficits in tau could have undesirable consequences. Mice that completely lack tau have normal learning and memory and cognition [191, 230, 275], with a minor, variable motor phenotype in later life [186, 191, 230, 330]. On the other hand, it is important to note, that tau deletion was shown to be associated with brain iron accumulation, brain insulin resistance and deficits in synaptic plasticity and cognition [6, 185, 218]. However, observations in knockout models could be hampered by developmental compensation by other MT-associated proteins such as *MAP1B* [134, 318] and it is thus crucial to understand the consequences of tau knockdown, post-development, in the adult brain. In one recent study, bilateral hippocampal shRNA mediated knockdown of tau in adult mice caused significantly impaired motor coordination and spatial memory accompanied by reduced synaptic markers and dendritic spine density. Behavioural deficits were restored once tau repression was removed [332]. However, in other studies, generalised knockdown of CNS tau in adult mice caused no deviations in normal sensory, motor or cognitive tasks [82]. Based on these mixed findings, it would be important to ascertain the tolerability of different levels of tau knockdown – it is perceivable that partial knockdown of tau in the adult brain could be beneficial, and yet minimising any undesirable effects.

## Anti-aggregation agents

Hyperphosphorylated and truncated tau protein is susceptible to aggregation and loss of cytoskeletal microtubule-stabilizing properties, leading to neuronal damage and cell death. Compounds able to prevent aggregation may represent a promising strategy for effective treatment of Alzheimer’s disease [162, 356]. Two major approaches focus on phosphorylation of tau and prevention of tau oligomerization. The former involves the search for inhibitors of kinases which phosphorylate tau or phosphatase activators which dephosphorylate the protein [5, 189]. The latter seeks direct inhibitors of the tau aggregation process.

### Regulation of tau phosphorylation

Phosphorylation of tau is under tight control of various protein kinases and phosphatases [5, 189]. Among them, glycogen synthase kinase 3β (GSK-3β) and phosphatase 2A (PP2A) are two key enzymes involved in regulation of the phosphorylation state of tau. GSK-3β is a multitasking serine/threonine kinase largely expressed in CNS that phosphorylates tau mainly at the Ser199, Ser396 and Ser413 sites [16]. Furthermore, it has been shown that an increase in GSK-3β activity induces Αβ formation and is also implicated in other processes, including neuroinflammation and apoptosis [51]. Therefore, GSK-3β is validated as a therapeutic target for AD, and several chemical classes of GSK-3β inhibitors have been discovered and developed in preclinical [217, 253] or even clinical trials. Tideglusib (NP031112, NP-12), is an ATP non-competitive GSK-3β inhibitor demonstrated to reduce spatial memory deficits in transgenic mice in preclinical studies [76]. While it has reached clinical trials, no satisfactory therapeutic results were obtained during phase II.

Tau phosphorylation is also regulated by O-GlcNAcylation, a non-canonical glycosylation involving the attachment of single O-linked N-acetylglucosamine (O-GlcNAc) moieties to serine and threonine residues [376]. O-GlcNAcylation is regulated by two enzymes, O-GlcNAc transferase catalyzing the transfer of GlcNAc to proteins, and N-acetylglucosaminidase (OGA) catalyzing the removal of GlcNAc from proteins [377]. Thiamet-G – a potent OGA inhibitor, that can influence O-GlcNAc levels in the brain, reduced tau phosphorylation in the brain after intraventricular administration [377]. This finding was successfully replicated in additional study, where Thiamet-G prevented the progression of hyperactivity, slowed brain atrophy, and reduced brain hyperphosphorylated tau in tau transgenic model TG4510 [345]. Similarly, ASN120290 – a small molecule that inhibits O-GlcNAcase reduced tau phosphorylation and the number of neurofibrillary pathology in the brain of transgenic mice P301L. ASN120290 which received Orphan Drug Designation from the Food and Drug Administration (FDA) for PSP, has already initiated Phase I clinical trials.

### Inhibition of tau aggregation

The most common direct inhibitor of tau protein aggregation is methylene blue (MB), which belongs to the class of thiazine dyes. Methylene blue, also known as methylthionine chloride (MTC), was originally synthesized at the end of the nineteenth century and used to treat malaria. It later found use as an antibacterial, antiviral and anticancer agent, applied in the treatment of various disorders. It is worth noting that its structure has played an important role in the development of phenothiazine-like compounds, including antipsychotic and antihistamine drugs. The anti-aggregating effect of phenothiazines upon the tau protein, discovered by Wischik and co-workers [355] over twenty years ago, paved the way for a new class of for potential anti-AD agents. However, during this time, most researchers focused on β-amyloid targets, and over the next two decades few achievements concerning tau were reported. Nonetheless, several chemical classes of tau aggregation inhibitors have been synthesized and presented [47, 48].

The tau aggregation inhibitor, methylene blue, occurs in two main forms, which are in equilibrium, depending on the redox potential of the solution. The first oxidized cationic state is characterized by a dark blue color, while the second (reduced form) is colorless and also referred to as leucomethylene blue (leuco-methylthioninum, LMT). Structurally, MTC is an aromatic anthracene compound (polyaromatic), whereas LMT is classified as a phenothiazine. It has been shown that anthracene-type compounds inhibit the tau protein, while phenothiazines, with nonaromatic tricyclic structures are inactive in this respect. MTC acts as a prodrug, and in acidic pH converts into leuco-methylthioninium, which can penetrate the BBB and reach brain tissues [17]. Many studies have demonstrated that MTC has a broad spectrum of pharmacological activity [251, 319]. The inhibition of tau aggregation by MTC has been confirmed by numerous in vitro tests as well as in in vivo models in transgenic mice. The dye has properties which inhibit microtubule assembly, prevent tau interaction, inhibit β-amyloid aggregation as well as α-synuclein aggregation. MTC counteracts mitochondrial damage caused by oxidative stress; it also has a positive effect on regulation of autophagy, acetylcholine E (AchE) inhibition, monoamine oxidases, the glutamatergic system and inhibition of noradrenaline uptake. From the point of view of potential clinical applications, the most important properties of MTC include: inhibition of microtubule formation, improvement of mitochondrial oxidation and inhibition of monoamine oxidase A [239].

In clinical trials, MTC was introduced under the name Rember™ (TauRx Therapeutics) as a potential anti-AD drug candidate. Some improvements in AD-related symptoms have been reported, but the drug failed phase II trials due to undesirable side effects, including diarrhoea, urgency, painful urination, dizziness and others (Clinical Trial Identifier, NCT00515333 and NCT00684944). Results of these studies prompted researchers to develop a new generation of MTC derivatives. These new compounds (LMTX) include leuco-methylthionium bis (hydro-methanesulfonate (LMTM) and leuco-methylthionium dihydrobromide (LMTB) – stable, reduced forms that permit direct absorption of LMT without the need for the aforementioned conversion step (Fig. 3).

LMTM (TRx0237) has reached phase III trials, and was better absorbed, with improved safety and tolerability compared to methylene blue (Rember™). Nevertheless, results of Phase III clinical trials involving LMTM in the treatment of AD were disappointing as they did not yield unambiguously positive data. The first phase III trial (NCT01689246) included 891 participants with mild to moderate AD, who received 125 mg of LMTM twice a day, or 75 mg twice a day while the control group received 4 mg twice a day. No significant difference in cognitive faculties or the ability to perform daily activities was observed between the treatment and control groups [110]. Due to the low number of participants (79) in this study, these results require further confirmation. Currently, TauRx has begun a new clinical trial (LUCIDUTY, NCT03446001) using FDG-PET imaging to examine the potential of LMTX in delaying the progression of pathological changes in the brain in AD patients who do not receive cholinesterase inhibitors or memantine. This trial is aimed at patients with early AD, with treatment lasting for 9 months (at doses of 8 mg/day and 16 mg/day). Thus, LMTM is being developed as an anti-AD treatment option based on inhibition of tau aggregation. Moreover, LMTC has demonstrated amelioration of α-synuclein pathology in a transgenic mouse model of synucleinopathy, and may therefore find use as a potential disease modification therapy in Parkinson’s disease (PD) and other synucleinopathies [290].

Since the discovery of the tau aggregation inhibitory activity of methylene blue, several chemical classes of compounds have been identified. These include derivatives of phenothiazines, polyphenols, benzothiazoles and porphyrins [319]. It has been observed that all these tested derivatives inhibited both tau filament formation and Aβ fibril formation. Further research carried out by Bulic and E. Mandelkow [47, 48], based on screening of a random library of 200,000 compounds, led to the identification of new chemical structures for potential tau inhibitors, including rhodamines, phenylthiazolyl-hydrazides, *N*-phenylamines, anthraquinones, benzothiazoles. Using quantitative high-throughput screening, Crowe and co-workers [70] discovered that aminothienopyrydazines (AZPZs) also inhibit of tau assembly.

Another potential source of anti-aggregation agents is provided by the multi-target-directed ligand approach. This strategy is suitable for complex diseases such as Alzheimer’s disease [18, 83, 264]. Therefore, many multifunctional compounds have been obtained by combining various pharmacophores targeting neurodegenerative processes into a single molecule. Among them multimodal molecules have been discovered that are endowed with tau aggregation inhibitory activity as well as other desirable properties. Selected examples of multifunctional agents are presented below.

**Fig. 4:**
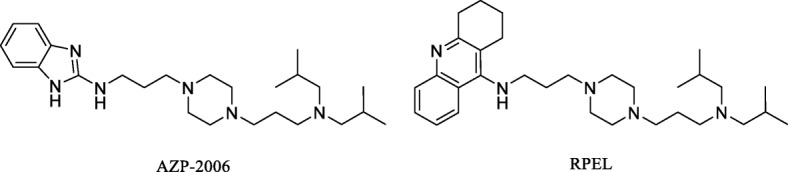
Multifunctional derivatives of piperazine

Compound AZP2006, an *N,N′*-disubstituted piperazine [226, 297], reduces the release of Aβ species and targets both amyloid and tau pathologies. It was demonstrated to improve cognitive faculties in various mouse models of both amyloid and tau pathology [21]. AZP2006 underwent phase I clinical trials on AD, and has now been classed as an orphan drug for the treatment of progressive supranuclear palsy (PSP). Another new compound, named RPEL, is a piperazine derivative that contains the pharmacophore fragment of tacrine [226] (Fig. 4). This dual-action compound showed inhibitory potency against cholinesterase (IC_50_ *h*AChE = 0.8 nM), reduced the phosphorylation of tau protein and inhibited the release of the Aβ peptide. Moreover, it displayed in vivo potency in transgenic mouse models and reduced memory loss.

**Fig. 5:**
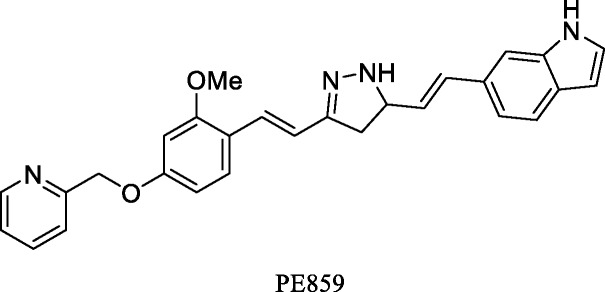
Structure of curcumin derivative PE859 dual tau and β-amyloid inhibitor

Japanese researchers [246, 247] presented a new tau inhibitor compound, PE859, based on the curcumin structure (Fig. 5). Promising results were obtained in both in vitro and in vivo studies – the compound was shown to counteract tau aggregation and prevent the onset and progression of nerve dysfunction in an in vivo model. Furthermore, it inhibits both tau and Aβ aggregation and alleviates cognitive dysfunction in vivo.

**Fig. 6:**
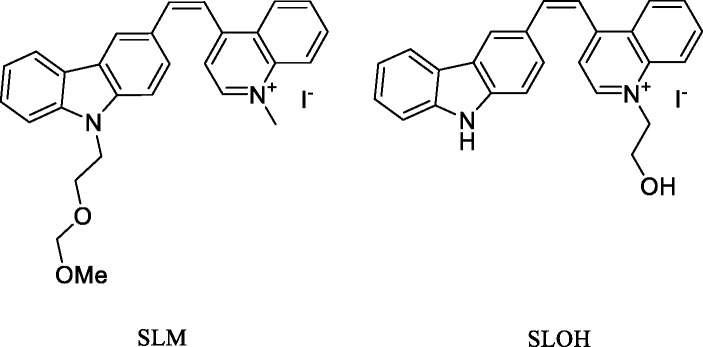
Structure of multifunctional carbazole–based cyanine compounds

Two carbazole-based cyanine compounds named SLM and SLOH were described as strong inhibitors of Aβ aggregation in vitro and were able to alleviate the pathological symptoms and memory deterioration in AD model mice [364–366] (Fig. 6). These multifunctional compounds also reduced tau hyperphosphorylation as well as significantly attenuated neuroinflammation through inhibitiion of GSK-3β activity. They showed a good pharmacokinetic profile, with high BBB permeability, which justifies their further development as AD drug candidates [379].

Dual inhibitors acting against β-secretase (BACE1) and glycogen synthase kinase 3β (GSK-3β), with well-balanced in vitro activity (in μM range), were synthesized in the class of triazinone derivatives [265]. These compounds displayed strong neuroprotective and neurogenic effects, and also showed good BBB permeability in a pharmacokinetic evaluation in mice. A new multi-target strategy for designing anti-AD agents involves compounds which combine GSK-3β and tau aggregation inhibitors [109]. Derivatives of 2,4-thiazolidinedione showed activity against GSK-3β (at micromolar IC_50_ values) and were also found to inhibit tau aggregation. Other examples of multifunctional compounds include rhein-huprine hybrids, which showed AChE and BACE1 inhibitory activity, as well as Aβ_1-42_ and tau anti-aggregating properties [259]. A 1-benzylamino-2-hydroxyalkyl derivative with a diphenylpiperazine fragment, selected form a series of compounds, showed balanced inhibitory activity against both disease-modifying targets, inhibition of BACE1, inhibition of Aβ, inhibition of tau aggregation, as well as inhibition of BuChE as a symptomatic target [254]. Jiang and co-workers [158] described a new class of dual GSK-3β and AChE inhibitors. These multifunctional compounds were designed by incorporating a tacrine fragment at the thiazolyl ring, as the pharmacophore responsible for GSK-3β inhibition. The resulting derivatives were very potent inhibitors of both targets (in the nanomolar range). The most promising compound from this series significantly inhibited tau protein phosphorylation and counteracted self-aggregation of Aβ_1-42_. In addition, it was not toxic and proved effective in an in vivo assay in mice, by significantly improving memory.

Most of the above-described direct tau inhibitors and multifunctional compounds have shown activity in in vitro tests, but only some of them have been evaluated in vivo in extended pharmacological, preclinical studies. Moreover, it is difficult to predict further development of these compounds. Due to the complex nature of AD, it seems reasonable to pursue development of combination therapies, as well as new alternative approaches which involve multi-target drugs. It is probable that a molecule capable of acting on two recognized targets, with one of them belonging to the tau cascade, might bring clinical benefits compared to drugs which only address only specific target.

## Concluding remarks and future directions

Tau is a multifaceted protein with a plethora of physiological functions. In the disease condition, tau protein drives neurodegeneration and causes neurodegenerative disorders such as Alzheimer’s disease. Pathologically modified tau has become an important therapeutic target for AD and related tauopathies. Although no disease-modifying treatments are yet available, many new therapeutic approaches targeting pathological forms of tau are being tested in clinical trials. Disease-modifying therapy is aimed at preventing, slowing or ameliorating the production, oligomerisation, aggregation and deposition of pathological tau protein. The most promising therapeutic strategies include active tau vaccines and therapeutic monoclonal antibodies. Besides immunotherapy, there are many other therapies currently being explored in the treatment of tau neurodegeneration such as modulation of tau phosphorylation, inhibition of tau aggregation or regulation of its expression. While waiting for the results of ongoing clinical trials, we can continue to unravel the complexities of tau proteome and different biological functions of this peculiar brain protein.

## Abbreviations

1 N: First insert
2 N: Second insert
3R: Three repeat
4R: Four repeat
Aβ: β-amyloid
aa: Amino acids
AAV: Adeno-associated virus
AChE: Acetylcholine E
AD: Alzheimer’s disease
AGD: Argyrophilic grain disease
ApoE4: Apolipoprotein E4
APP: Amyloid precursor protein
ARTAG: Age-related tau astrogliopathy
ASO: Antisense oligonucleotide
BBB: Blood-brain barrier
BIN1: Myc box-dependent-interacting protein 1
CDB: Corticobasal degeneration
CDRs: Complementarity determining regions
CNS: Central nervous system
CSF: Cerebrospinal fluid
C-terminal: Carboxy-terminal
DMD: Duchenne muscular dystrophy
EOAD: Early-onset Alzheimer’s disease
FDA: Food and Drug Administration
FDG: Fluorodeoxyglucose
FGF-2: Fibroblast growth factor 2
FTD: Frontotemporal dementia
FTLD: Frontotemporal lobar degeneration
FTP: [18F] Flortaucipir
GGT: Globular glial tauopathy
GSK-3β: Glycogen synthase kinase 3β
HD: Huntington’s disease
HMW: High molecular weight tau
*HTT*: Huntingtin gene
IRS-1: Insulin receptor substrate 1
K_a_: Association constant
K_d_: Dissociation constant
KLH: Keyhole limpet haemocyanin
LMT: Leuco-methylthioninum
LMTB: Leuco-methylthionium dihydrobromide
LMTM: Leuco-methylthionium bis(hydro-methanesulfonate
lncRNA: Long non-coding RNA
LOAD: Late- onset Alzheimer’s disease
MAO-B: Monoaminoxidase B
MAP: Microtubule-associated protein
MB: Methylene blue
MIR: Mammalian interspersed repeat
MRI: Magnetic resonance imaging
mRNA: Messenger RNA
MT: Microtubules
MTBR: Microtubule binding region
MTC: Methylthionine chloride
MTL: Medial temporal lobe
NATs: Natural antisense transcripts
NFT: Neurofibrillary tangles
NPC: Nuclear pore complex
N-terminal: Amino-terminal
O-GlcNAc: O-linked N-acetylglucosamine
PART: Primary age-related tauopathy
PET: Positron emission tomography
PHF: Paired-helical filaments
PiD: Pick’s disease
PP2A: Phosphatase 2A
PSP: Progressive supranuclear palsy
PTEN: Phosphatase and tensin homolog
PTM: Post translational modification
RISC: RNA-induced silencing complex
RNAi: RNA interference
SMA: Spinal muscular atrophy
SMaRT: Spliceosome-mediated *trans-*splicing
SMN: Survival motor neuron
SPR: Surface plasmon resonance spectroscopy
SPs: Senile plaques
TRIM21: Tripartite motif-containing protein 21
